# Neuromyelitis Optica Spectrum Disorder Following Pituitary Tumor Resection: A Rare but Critical Differential in Neurosurgical Practice

**DOI:** 10.1002/ccr3.72988

**Published:** 2026-06-18

**Authors:** Arad Iranmehr, Mohammad Shirani, Abdorreza Naser Moghadasi, Shirin Irani, Narges Akhlaghi, Samira Mirzaei, Hesam Ghadirian

**Affiliations:** ^1^ Department of Neurosurgery, Sina Hospital Tehran University of Medical Sciences Tehran Iran; ^2^ Multiple Sclerosis Research Center, Neuroscience Institute Tehran University of Medical Sciences Tehran Iran; ^3^ Otorhinolaryngology Research Center, Amir‐Alam Hospital Tehran University of Medical Sciences Tehran Iran; ^4^ Department of Pathology, Sina Hospital Tehran University of Medical Sciences Tehran Iran; ^5^ Department of Radiology, Sina Hospital Tehran University of Medical Sciences Tehran Iran

**Keywords:** adenoma, demyelination, neuromyelitis optica, pituitary

## Abstract

Neuromyelitis optica spectrum disorder (NMOSD) is a rare, immune‐mediated astrocytopathy disorder that predominantly affects adults. Postoperative cases are scarcely reported. A 75‐year‐old woman underwent uncomplicated endoscopic resection of a nonfunctional pituitary macro‐adenoma. Seven days postoperatively, she developed encephalopathy without focal deficits. Magnetic resonance imaging (MRI) revealed extensive confluent T_2_ and fluid attenuated inversion recovery (FLAIR) hyperintensities in the corpus callosum, periventricular white matter, and cervical spinal cord, absent preoperatively. Metabolic, infectious, and structural etiologies were excluded. Despite high‐dose corticosteroids and intravenous immunoglobulin (IVIG) administration, she deteriorated with brainstem involvement, respiratory failure, and fatal arrhythmias. This exceptionally rare case emphasizes NMOSD as a critical differential in postoperative neurological decline, particularly after CNS procedures. Clinicians must prioritize early recognition to guide timely intervention. Future studies should investigate surgical stress, pituitary dysfunction, and immune activation in demyelination pathogenesis.

## Key Clinical Message

Transsphenoidal surgery may be a provocative factor to induce the demyelinating process in patients without any previous history.

## 1 Introduction

Neuromyelitis optica spectrum disorder (NMOSD) represents a neuroimmune‐mediated astrocytopathy condition characterized by selective vulnerability to optic nerve and spinal cord inflammation within the central nervous system (CNS) [1, 2]. The presence of hypothalamic hypophysis involvement in cases with NMOSD has been reported in previous literature, and in some of these cases, hypophyseal and hypothalamic enhancement with gadolinium on dedicated MRI images of the pituitary was also described [2]. The coexistence of pituitary adenomas with CNS disorders, particularly immune‐mediated conditions, remains a rare and understudied phenomenon. While pituitary adenomas are well‐documented for their hormonal and compressive effects, emerging reports suggest potential immunological interactions, such as antigen exposure during surgical interventions or molecular mimicry, which may trigger autoimmune responses [3, 4].

This case report delineates the rare coexistence of NMOSD and a nonfunctional pituitary adenoma in a single patient. It highlights the challenges in diagnosing coexisting CNS disorders and prompts further investigation into possible immune‐related or disease‐related connections between myelin sheath damage and pituitary tumors.

## 2 Case History / Examination

A 75‐year‐old woman presented with a 1‐year history of visual field impairment that had a progressive course and was worse in her left eye, and no problems were found on ophthalmic examination. Imaging revealed a sellar/supra‐sellar mass exerting compressive effects on the optic chiasm (Figure 1A–F). Hormonal profiling confirmed a nonfunctional pituitary adenoma. The patient underwent uncomplicated endoscopic endonasal trans‐sphenoidal resection of the adenoma. Intraoperatively, a minor arachnoid layer breach resulted in cerebrospinal fluid (CSF) leakage, which was repaired using periumbilical fat grafting.

**FIGURE 1:**
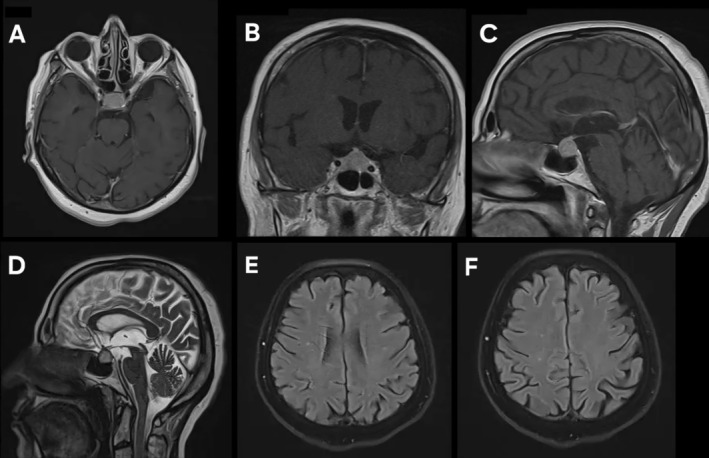
Preoperative magnetic resonance imaging (MRI). (A–C) Axial, coronal, and sagittal T_1_ images with Gadolinium (GAD) injection show macro‐adenoma with homogenous enhancement in the sellar and supra‐sellar space with compression effect on the optic chiasm from the inferior surface. (D) Sagittal T_2_ MRI shows normal corpus callosum and upper cervical cord, without any abnormal signal in the cord. (E, F) Axial fluid attenuated inversion recovery (FLAIR) images from centrum semiovale and corpus callosum show no specific lesion.

Hormonal replacement therapy (glucocorticoids and thyroid hormone) was initiated after surgery. During the early postoperative period, the patient developed transient diabetes insipidus, successfully managed with sublingual desmopressin. Histopathologic examination report was pituitary neuroendocrine tumor (Figure 2A–C). There was no special problem in the first 6 days after surgery but on the seventh day, when the patient was ready to be discharged, abruptly she experienced progressive loss of consciousness without focal neurological deficits.

**FIGURE 2:**
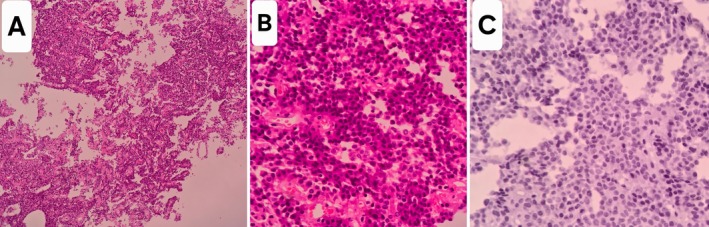
Pituitary neuroendocrine tumor (Pit‐NET) histology. (A) Neoplastic tissue composed of sheets of tumoral cells, some form pseudo‐rosettes around vascular channels (Hematoxylin and Eosin, X40); (B) Tumoral cells have uniform nuclear morphology with stippled chromatin, inconspicuous nucleoli, and moderately abundant cytoplasm (Hematoxylin and Eosin, X100); (C) Immunostaining for CD_138_ showed no cytoplasmic reactivity (X100).

## 3 Differential Diagnosis, Investigation and Treatment

Emergent evaluations to rule out hydrocephalus, meningitis, and hypothyroidism (CT scan of the brain, lumbar puncture, and hormonal assays respectively) were carried out but no explanation was found. Brain MRI with diffusion‐weighted imaging (DWI) appeared normal, but FLAIR sequences demonstrated new multifocal hyperintense lesions within the corpus callosum and periventricular white matter that were absent in preoperative images. Subsequent gadolinium‐enhanced MRI of the brain and spine revealed extensive, confluent, and enhancing demyelinating lesions involving the cervical spinal cord, medulla, and cerebral white matter, accompanied by significant edema of medulla (Figure 3A–G). Based on neurological consultation, the diagnosis of a demyelinating process was established and high‐dose intravenous methylprednisolone (1 g/day for 5 days) followed by intravenous immunoglobulin (0.4 g/kg/day for 5 days) was injected to her. The serum investigation was positive for aquaporin 4 antibody (AQP4). Despite therapy, she developed respiratory failure and cardiac rhythm disturbances, necessitating intubation. The patient exhibited no clinical improvement and unfortunately died after 12 days.

**FIGURE 3:**
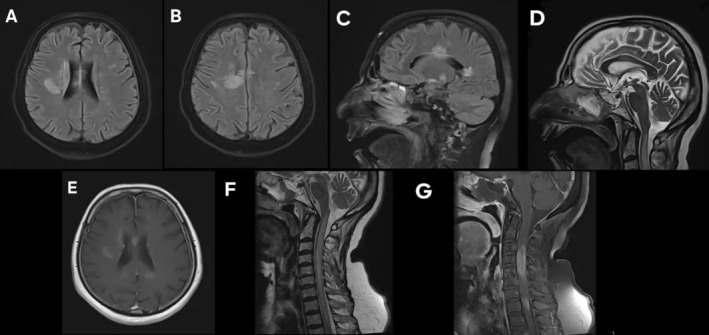
Postoperative MRI. (A, B) Axial FLAIR, (C) Sagittal FLAIR, (D) Sagittal T_2_ and (E) Axial T_1_ with GAD injection brain MRI show multiple signal changes in the periventricular white matter and corpus callosum with avid enhancement. (F) Sagittal T_2_ and G. Sagittal T_1_ with GAD injection cervical MRI shows diffuse edema that extends from medulla to C_7_ and enhancement of demyelinating plaques.

## 4 Discussion

Two distinct mechanisms may explain the development of autoimmune demyelination disorders following surgical procedures. First, autoimmune activation could occur when surgical stress or tissue damage releases CNS antigens, prompting a T‐cell‐mediated immune attack on myelin, as in the autoimmune response seen in postinfectious ADEM [5, 6]. Second, molecular mimicry might play a role if foreign antigens introduced during surgery—such as those from instruments, implants, or contrast agents—structurally resemble myelin proteins, thereby triggering cross‐reactive immune responses that inadvertently target the body's tissues [7, 8, 9, 10]. Both pathways highlight how surgical interventions, despite their therapeutic intent, may paradoxically incite immune‐mediated demyelination through the disruption of immunological tolerance.

Previous studies have documented acute disseminated encephalomyelitis (ADEM) following surgical interventions [11, 12, 13]. ADEM has been observed in neurosurgical contexts after procedures such as endoscopic colloid cyst removal [14], transnasal endoscopic craniopharyngioma resection [7], and cerebral aneurysm coiling [12]. Notably, all three documented cases involved the utilization of endoscopic or endovascular techniques to address CNS pathologies. This recurrent association raises critical questions regarding the potential role of procedural factors, such as tissue manipulation or biomaterial exposure, in triggering immune‐mediated demyelination.

For instance, recent literature highlights cases of postoperative acute disseminated encephalomyelitis (ADEM) following pituitary tumor resection, implicating surgical stress in disrupting immune tolerance [7]. ADEM constitutes a monophasic, immune‐driven neuroinflammatory condition marked by acute demyelination within CNS. Clinically, it presents with rapid‐onset encephalopathy, multifocal neurological dysfunction, and in severe cases, mortality. As a member of the acquired demyelination syndromes, ADEM is distinguished by its temporal association with antecedent immunological triggers, most commonly viral infections (50%–75% of cases) or vaccinations, with bacterial etiologies accounting for a minority (~10%–15%) [7, 15]. Pathophysiological models posit molecular mimicry as the dominant mechanism, wherein cross‐reactive T‐cell responses erroneously target epitopes shared between microbial antigens and CNS myelin proteins (e.g., myelin oligodendrocyte glycoprotein) [5]. While pediatric predominance is well‐documented, adult‐onset ADEM remains a rare condition. This disparity has precluded consensus on standardized diagnostic criteria for adults, posing challenges in differential diagnosis (e.g., distinguishing ADEM from multiple sclerosis) and hindering multicenter research efforts [7, 9].

Similarly, optic neuritis—a hallmark of NMOSD—has been sporadically linked to pituitary adenomas, raising questions about shared pathophysiological mechanisms, such as prolactin‐mediated oligodendrocyte dysregulation or hormonal‐immune cross‐talk [16]. These observations underscore the need to explore bidirectional relationships between pituitary pathology and neuro‐inflammatory disorders, particularly in viral infections like COVID‐19, which may exacerbate immune dysregulation [4, 16].

A documented case involving hypophyseal pathology (Craniopharyngioma) parallels the present clinical scenario [7], Emerging evidence indicates that prolactin mediates the proliferation of oligodendrocyte precursor cells (OPCs), thereby facilitating myelination processes. However, the precise molecular mechanisms governing OPC expansion and their differentiation into functional myelinating oligodendrocytes remain incompletely elucidated [17, 18, 19]. Preclinical investigations have revealed that prolactin signaling modulates OPC proliferation during pregnancy, enhancing axonal remyelination within the maternal CNS [19]. These findings propose potential therapeutic implications for prolactin, which may replicate pregnancy‐associated neuroprotective effects by stimulating OPC proliferation—critical for CNS myelin repair—in the context of demyelinating pathologies [17, 20]. This mechanism may be another possible explanation that should be investigated in the future in patients with pituitary pathology and demyelinating CNS disorders. The main limitations of this study are the single‐case nature of the report and the lack of follow‐up due to the patient's death. Further studies with larger cohorts are needed.

## 5 Conclusion

This report presents the first documented new‐onset NMOSD following pituitary adenoma resection, highlighting its infrequent occurrence and diagnostic complexity. The temporal proximity to surgery reinforces surgical triggers (e.g., antigen exposure) in immune‐mediated demyelination. Despite aggressive therapies, the fatal outcome emphasizes the critical need for improved management strategies. This unprecedented coexistence of NMOSD and pituitary pathology underscores potential CNS immune dysregulation mechanisms, urging further research. Clinicians must recognize this potential complication post‐CNS procedures, as early detection could alter prognosis in similarly rare, high‐risk scenarios.

## Author Contributions

**Arad Iranmehr:** conceptualization, investigation, methodology. **Mohammad Shirani:** conceptualization, visualization, writing – review and editing. **Abdorreza Naser Moghadasi:** conceptualization, investigation, methodology, project administration. **Shirin Irani:** conceptualization, investigation, methodology, project administration. **Narges Akhlaghi:** conceptualization, investigation, methodology, project administration. **Samira Mirzaei:** funding acquisition, methodology, validation, visualization. **Hesam Ghadirian:** conceptualization, methodology, project administration, resources, visualization, writing – original draft.

## Funding

The authors have nothing to report.

## Consent

The authors have obtained written informed consent to publish the details from the affected individuals.

## Conflicts of Interest

The authors declare no conflicts of interest.

## Data Availability

All the data used in this manuscript is available upon the request from corresponding author.

## References

[1] M. M. Al‐Salihi , M. M. Rahman , M. S. Al‐Jebur , et al., “Optic Neuritis Concomitant With Pituitary Macroadenoma in a Patient With Active COVID‐19 Infection: A Case Report,” International Journal of Surgery Open 35 (2021): 100390, 10.1016/j.ijso.2021.100390. 34568623 PMC8384762

[2] E. Brusse and C. Tijssen , “Neuromyelitis Optica With Endocrinopathy: Further Evidence of a New Syndrome,” Neuro‐Ophthalmology 25 (2001): 151–155, 10.1076/noph.25.3.151.7133.

[3] J. Honegger , M. Buchfelder , U. Schrell , E. F. Adams , and R. Fahlbusch , “The Coexistence of Pituitary Adenomas and Meningiomas: Three Case Reports and a Review of the Literature,” British Journal of Neurosurgery 3 (1989): 59–69, 10.3109/02688698909001027. 2675917

[4] W. Lu , Y. Shengkai , W. Yu , L. Aimin , Y. Shiwei , and X. Kang , “Case Report: Clinical Report of Co‐Occurrence of Pituitary Adenoma and Meningioma in the Sellar Region After Meningioma Treatment,” Frontiers in Neurology 13 (2022): 1042106, 10.3389/fneur.2022.1042106. 36561304 PMC9763986

[5] R. Paolilo , K. Deiva , R. Neuteboom , K. Rostásy , and M. Lim , “Acute Disseminated Encephalomyelitis: Current Perspectives,” Children (Basel) 7 (2020): 210. 33153097 10.3390/children7110210 PMC7692206

[6] K. Wucherpfennig and J. Strominger , “Molecular Mimicry in T Cell‐Mediated Autoimmunity: Viral Peptides Activate Human T Cell Clones Specific for Myelin Basic Protein,” Cell 80 (1995): 695–705, 10.1016/0092-8674(95)90348-8. 7534214 PMC7133435

[7] K. Gag , J. Müller , M. Süße , R. Fleischmann , and H. W. S. Schroeder , “Acute Disseminated Encephalomyelitis Following Endonasal Resection of a Craniopharyngioma: Illustrative Case,” Journal of Neurosurgery: Case Lessons 2 (2021): CASE21293, 10.3171/CASE21293. 35855278 PMC9281492

[8] Y. Katz‐Levy , K. Neville , and A. Girvin , “Endogenous Presentation of Self Myelin Epitopes by CNS‐Resident APCs in Theiler's Virus‐Infected Mice,” Journal of Clinical Investigation 104 (1999): 599–610, 10.1172/JCI7292. 10487774 PMC408545

[9] J. Murthy , “Acute Disseminated Encephalomyelitis,” Neurology India 50 (2002): 238–243. 12391446

[10] D. Pohl , G. Alper , and K. Van Haren , “Acute Disseminated Encephalomyelitis: Updates on an Inflammatory CNS Syndrome,” Neurology 87 (2016): S38–S45, 10.1212/WNL.0000000000002825. 27572859

[11] B. Ceronie and O. Cockerell , “Acute Disseminated Encephalomyelitis in an Older Adult Following Prostate Resection,” eNeurologicalSci 14 (2018): 40–42, 10.1016/j.ensci.2018.11.006. 30619950 PMC6304343

[12] L. Deus‐Silva , C. Lum , C. De Meulemeester , and M. dos Santos , “Severe Aggressive Acute Disseminated Encephalomyelitis‐Like Reaction After Aneurysm Coiling,” Neurosurgery 66 (2010): E222–E223, 10.1227/01.NEU.0000362001.81386.BE. 20023529

[13] L. White , L. White , D. Baxter , and T. Melhuish , “Adult Onset Acute Disseminated Encephalomyelitis Following Appendicitis: A Case Report,” Journal of Neurology and Neuroscience 7 (2016): S3.

[14] N. Desse , A. Sellier , C. Bernard , and A. Dagain , “Fatal Acute Disseminated Encephalomyelitis (ADEM) After Third Ventricle Colloid Cyst Resection With Ultrasonic Aspirator During Neuroendoscopic Procedure,” Acta Neurochirurgica 160 (2018): 1789–1792, 10.1007/s00701-018-3631-8. 30058026

[15] D. Koelman and F. Mateen , “Acute Disseminated Encephalomyelitis: Current Controversies in Diagnosis and Outcome,” Journal of Neurology 262 (2015): 2013–2024, 10.1007/s00415-015-7694-7. 25761377

[16] S. Ren , Q. Lu , Y. Xiao , et al., “Coexistence of Pituitary Adenoma and Primary Pituitary Lymphoma: A Case Report and Review of the Literature,” Frontiers in Surgery 9 (2022): 842830, 10.3389/fsurg.2022.842830. 35372490 PMC8966133

[17] M. Etemadifar , M. A. Najafi , M. R. Najafi , et al., “Multiple Sclerosis and Hyperprolactinemia: A Case–Control Study,” Acta Neurologica Belgica 115 (2015): 253–257, 10.1007/s13760-014-0403-y. 25501283

[18] J. M. Gensert and J. E. Goldman , “Endogenous Progenitors Remyelinate Demyelinated Axons in the Adult CNS,” Neuron 19 (1997): 197–203, 10.1016/s0896-6273(00)80359-1. 9247275

[19] C. Gregg , V. Shikar , P. Larsen , et al., “White Matter Plasticity and Enhanced Remyelination in the Maternal CNS,” Journal of Neuroscience: The Official Journal of the Society for Neuroscience 27 (2007): 1812–1823, 10.1523/jneurosci.4441-06.2007. 17314279 PMC6673564

[20] T. Moreau , S. Brunot , G. Couvreur , and A. Fromont , “Pregnancy and Multiple Sclerosis,” Presse Médicale (Paris, France: 1983) 39 (2010): 389–394, 10.1016/j.lpm.2009.07.028. 20116203

